# The Common Mechanisms of Sarcopenia and NAFLD

**DOI:** 10.1155/2017/6297651

**Published:** 2017-12-13

**Authors:** Yu Zhai, Qian Xiao

**Affiliations:** Department of Geriatrics, The First Affiliated Hospital of Chongqing Medical University, Chongqing 400016, China

## Abstract

Current studies have shown that sarcopenia and nonalcoholic fatty liver disease (NAFLD) have similar pathophysiological profiles. The cooccurrence of sarcopenia and NAFLD has been observed in elderly patients. The actions of these conditions are linked, and their treatments are similar. Therefore, studies should be conducted on NAFLD-sarcopenia rather than on NAFLD or sarcopenia.

## 1. Introduction

Many challenges have resulted from the ageing of society, and several growing health problems related to ageing need to be addressed by geriatric researchers, including sarcopenia, which is derived from the Greek term for loss of flesh and was first suggested by Rosenberg in 1989 [1]. Due to an increasing number of basic and clinical studies, the definition of sarcopenia has been refined. In 2010, the European Working Group on Sarcopenia in Older People (EWGSOP) reported sarcopenia as a syndrome characterised by progressive and generalised loss of skeletal muscle mass and strength with a risk of adverse outcomes such as physical disability, poor quality of life, and death [2].

Nonalcoholic fatty liver disease (NAFLD) is a genetic-environment-metabolic stress-related disease and has replaced viral liver diseases as the most common liver disease worldwide. For example, the Third National Health and Nutrition Examination Survey in the United States showed that the prevalence rate of NAFLD was 19.0% [3]. The metabolically active organ triad comprised of the liver, adipose tissue, and skeletal muscle contributes to NAFLD. Metabolic syndrome, which often occurs in older individuals, consists of central obesity, dyslipidaemia, hyperglycaemia, and other features. NAFLD plays an important role in the occurrence and development of these components [4].

Sarcopenia has been generally accepted by researchers as a geriatric syndrome. The risk of developing NAFLD increases with age [5]. Due to their common pathological and physiological mechanisms, it has already been reviewed and this has been a listed online review by Bhanji et al. [6]. Hence reference to this review has to be acknowledged. NAFLD and sarcopenia are both a threat to the health of older individuals and share many features. A recent study in Japan showed that skeletal muscle mass index (SMI) and hepatic steatosis are negatively correlated among male type 2 diabetic patients [7]. The Fifth Korea National Health and Nutrition Examination Survey has indicated that a low SMI is associated with the risk of NAFLD independent of other well-known metabolic risk factors in both genders [8]. Many studies have linked the mechanisms of sarcopenia and NAFLD.

This review aims to report recent evidence concerning the links between sarcopenia and NAFLD.

## 2. Insulin Resistance

Skeletal muscle and the liver are target organs of insulin, which plays an important role in glucose metabolism. Muscle and hepatic glycogen both contribute to energy metabolism in the human body. Insulin resistance (IR) is a pathological condition in which cells fail to respond normally to the hormone insulin. According to recent studies, IR could lead to glucose metabolism disorders and may also be a key factor in the development of sarcopenia and NAFLD [9, 10].

Srikanthan et al., in the National Health and Nutrition Examination Survey III, found that sarcopenia, independent of obesity, is associated with adverse glucose metabolism, which suggests that diabetes might be linked to low muscle mass [11]. Studies have shown that IR participates in the development of sarcopenia [12, 13]. Insulin-mediated accretion of muscle mass has been attributed to the activation of mammalian target of rapamycin (mTOR) or ribosomal protein S6 kinase beta-1 (S6 K1) as well as stimulation of mRNA translation [14, 15]. Insulin also plays a clear role in reducing skeletal muscle protein breakdown [16]. When IR occurs in myocytes, the reduction of protein synthesis [17] and increase in protein catabolism [18] contribute to muscle mass loss, which results in sarcopenia.

Studies have also suggested that NAFLD is an outcome of IR. Insulin inhibits the accumulation of adipose tissue by lipolysis; therefore, nonesterified fatty acids decrease in the blood circulation, resulting in reduced flow into the liver. IR-mediated hyperinsulinaemia induces the upregulation of sterol regulatory element-binding protein 1c (SREBP-1c) and the inhibition of *β*-oxidation in the liver, resulting in free fatty acid and triglyceride accumulation [19]. Utzschneider and Kahn demonstrated that IR might enhance hepatic fat accumulation by increasing free fatty acid delivery, while hyperinsulinaemia stimulates anabolic processes. Moreover, the effects of weight loss, metformin, and thiazolidinediones, which are treatments aimed at improving insulin sensitivity, have been evaluated in patients with NAFLD, and these medications have shown some benefit [20].

## 3. Vitamin D

A geriatric study suggested that increased visceral fat and lower muscle mass are associated with lower vitamin D (VD) levels in elderly Korean men [21]. Scott et al. reported that VD may be important for the maintenance of muscle function and greater skeletal muscle mass [22]. The results of a prospective, population-based study also showed that lower VD levels increase the risk of sarcopenia in older men and women [23]. VD plays roles in the proliferation and differentiation of myoblasts, the production and growth of skeletal muscle cells, and the inflammation of skeletal muscle [24–26]. The nuclear vitamin D receptor (VDR), a ligand-activated transcription factor and a member of the superfamily of nuclear receptors for steroid hormones, has been identified in human skeletal muscle. VD, bound to its receptor, modulates the expression of genes related to the regulation of cell proliferation and differentiation [27]. However, an increasing deficiency of VD and decreasing expression of VDR occur with ageing [28], which may lead to sarcopenia, and thus, individuals with sarcopenia have lower VD levels [29]. In practice, VD supplementation increases the expression of VDR in skeletal muscle, which blocks the development of sarcopenia [30].

The association between VD levels and NAFLD has been recognised. A meta-analysis that evaluated 17 cross-sectional and case-control studies indicated that NAFLD patients have decreased serum VD concentrations [31]. Low VD levels were strongly associated with the presence of NAFLD in an adult population with normal serum liver enzymes, independent from metabolic syndrome, diabetes, and IR profile [28]. Furthermore, VD downregulated the expression of SREBP-1c mRNA and its target genes, acetyl-CoA carboxylase and fatty acid synthase, which are involved in lipogenesis. Peroxisome proliferator-activated receptor *α* and its target gene carnitine palmitoyltransferase-1, which are involved in hepatic fatty acid oxidation, were upregulated by VD [32]. Another study in obese rats found that VD deficiency exacerbates NAFLD through the activation of Toll-like receptors in the liver. VD deficiency causes IR, higher hepatic resistance gene expression, and upregulation of hepatic inflammatory and oxidative stress genes [33]. Recent data also show that VDR knockout mice spontaneously develop hepatic steatosis [34].

Presently, the majority of studies show that VD plays a role in sarcopenia and NAFLD. Only two studies have found no such relationship. One study observed no significant association between VD and NAFLD in a Chinese population [35], while the other, a Korean sarcopenic obesity study, showed that VD was not correlated with the SMI or liver attenuation index (LAI) [36].

## 4. Chronic Low-Grade Inflammation

Ageing is accompanied by inflammatory disorders, slight elevations in circulating proinflammatory mediators, and decreases in anti-inflammatory cytokines, which correspond to a chronic low-grade inflammatory profile (CLP). Sarcopenia and NAFLD could be linked by the CLP. Cesari et al. reported that C-reactive protein (CRP) and interleukin-6 (IL-6) are positively associated with total fat mass and negatively associated with appendicular lean mass [37]. A study by Hong et al. showed that sarcopenia patients have increased high-sensitivity CRP levels, which are correlated with the SMI and LAI [36]. Catabolic inflammatory processes enhance sarcopenia among older individuals, especially those with very advanced age. IL-6 and tumour necrosis factor-*α* (TNF-*α*) are the most commonly reported inflammatory markers in cross-sectional and longitudinal studies [38]. An increase in TNF-*α* could lead to a reduction in myoneme protein synthesis and promote the decomposition of striated muscle cells by regulating transcriptional factors. In the liver, TNF-*α* could promote lipid accumulation through activation of de novo fat synthesis. TNF-*α* also activates nuclear factor *κ*B, a central transcriptional factor for many proinflammatory cytokines, via TNF receptor 1. Increasing proinflammatory cytokines might contribute to the development of NAFLD and the catabolism of muscle [38, 39]. Numerous inflammatory mediators are released from immune cells during ageing, and adipocytes contribute to the development of IR [40], which is one of the main causes of both sarcopenia and NAFLD, as previously described.

## 5. Other Mechanisms

Myostatin (MSTN), a transforming growth factor beta family member, is produced and released by myocytes and is a potent negative regulator of skeletal muscle growth. It has been reported, albeit in abstract form only, that the MSTN receptor is present in hepatic stellate cells. This finding raises the question of whether NAFLD results in sarcopenia via activation of MSTN in skeletal muscle or whether sarcopenia is the primary abnormality related to MSTN and functions in an endocrine manner to activate fibrogenic hepatic stellate cells. Numerous animal studies have shown that MSTN exhibits significant hepatic effects by regulating skeletal muscle metabolism. Blocking MSTN increases muscle mass, protects mice from NAFLD, and improves insulin sensitivity [41, 42].

Adiponectin, a protein secreted by adipocytes, is involved in IR, disorders of substrate oxidation, and mitochondrial dysfunction in multiple organs. Two types of adiponectin receptors have been reported. Type 1 is highly expressed in skeletal muscle. Type 2 is mostly expressed in the liver. Reduced hepatic expression of the type 2 receptor might be of pathophysiological relevance for NAFLD [43]. The type 1 receptor is involved in regulating glucose levels and fatty acid breakdown. Some researchers have questioned whether crosstalk occurs between MSTN and adiponectin [44].

Perilipin 2, a protein associated with the metabolism of intracellular lipid droplets, has long been considered only to be involved in lipid storage. However, the expression of perilipin 2 affects the severity of a variety of metabolic and age-related diseases, including sarcopenia and NAFLD, and its downregulation in mice mitigates or prevents some of the above-mentioned diseases. In humans, high levels of perilipin 2 are present in patients with sarcopenia and hepatic steatosis [45].

A diet-induced NAFLD mouse model has shown that NAFLD is associated with sarcopenia, decreased muscle strength, and reduced insulin-like growth factor-1 (IGF-1) serum levels, suggesting that IGF-1 reduction may be involved in the pathogenesis of NAFLD-associated sarcopenia [46]. Recently, the growth hormone/IGF-1 axis has been postulated to play a role in linking NAFLD and low muscle mass. Impairment of this axis appears to be associated with the risk of development of sarcopenic obesity and ectopic fat deposition in the liver [47].

## 6. Conclusion

Current studies have shown that NAFLD and sarcopenia may share common pathophysiological mechanisms. Sarcopenia is independently associated with NAFLD [48]. Moreover, the cooccurrence of sarcopenia and NAFLD has been observed in elderly patients. There is even a previous study exploring the complex interrelationships between sarcopenia and nonalcoholic steatohepatitis [6]. However, the high global prevalence of NAFLD and the popularisation of ultrasonography and computed tomography contribute to the high detection rate of NAFLD, while the equipment for bioelectrical impedance analysis and dual energy X-ray absorptiometry is less commonly available. Furthermore, tests for gait speed and handgrip strength are not typically conducted, resulting in a lower detection rate of sarcopenia results and fewer studies focused on this condition. These two conditions should be addressed together rather than separately. Physical exercise and nutritional supplementation are the core strategies for the management of sarcopenia. The first line of treatment for NAFLD is usually a combination of a healthy diet and exercise. Due to the increasing availability of interventions, number of VD supplements, and incidence of insulin sensitisation, geriatric researchers should combine studies on NAFLD and sarcopenia. The clinical guidelines for treating these anomalies should address both conditions.
